# Pre-treatment expectations of patients with spinal metastases: what do we know and what can we learn from other disciplines? A systematic review of qualitative studies

**DOI:** 10.1186/s12885-020-07683-7

**Published:** 2020-12-09

**Authors:** R. Gal, D. Oostinga, H. Wessels, J. J. Verlaan, R. Charest-Morin, C. G. Fisher, H. M. Verkooijen, A. L. Versteeg

**Affiliations:** 1 Division of Imaging and Cancer, University Medical Center Utrecht, University of Utrecht, Heideberglaan 100, 3584 CG Utrecht, the Netherlands; 2 Department of Orthopaedic Surgery, University Medical Center Utrecht, University of Utrecht, Utrecht, the Netherlands; 3 Department of Corporate Communications, University Medical Center Utrecht, Utrecht, The Netherlands; 4 Department of Orthopaedics, Division of Spine, Vancouver General Hospital/University of British Columbia, Vancouver, British Columbia Canada

**Keywords:** Spinal metastases, Treatment outcomes, Patient expectations, Spine surgery, Advanced cancer

## Abstract

**Background:**

Little is known about treatment expectations of patients with spinal metastases undergoing radiotherapy and/or surgery. Assuming that patients with spinal metastases share characteristics with patients who had spinal surgery for non-cancer related conditions and with advanced cancer patients, we performed a systematic review to summarize the literature on patient expectations regarding treatment outcomes of spinal surgery and advanced cancer care.

**Methods:**

A comprehensive search was performed in MEDLINE, EMBASE and PsycINFO for studies between 2000 and sep-2019. Studies including adult patients (> 18 years), undergoing spinal surgery or receiving advanced cancer care, investigating patients’ pre-treatment expectations regarding treatment outcomes were included. Two independent reviewers screened titles, abstracts and full-texts, extracted data and assessed methodological quality.

**Results:**

The search identified 7343 articles, of which 92 were selected for full-text review. For this review, 31 articles were included. Patients undergoing spinal surgery had overly optimistic expectations regarding pain and symptom relief, they underestimated the probability of functional disability, and overestimated the probability of (complete) recovery and return to work. Studies highlighted that patients feel not adequately prepared for surgery in terms of post-treatment expectations. Similarly, advanced cancer patients receiving palliative treatment often had overly optimistic expectations regarding their survival probability and cure rates.

**Conclusions:**

Patients tend to have overly optimistic expectations regarding pain and symptom relief, recovery and prognosis following spinal surgery or advanced cancer care. Pretreatment consultation about the expected pain and symptom relief, recovery and prognosis may improve understanding of prognosis, and promote and manage expectations, which, in turn, may lead to better perceived outcomes.

**Trial registration:**

PROSPERO registration number: CRD42020145151.

**Supplementary Information:**

The online version contains supplementary material available at 10.1186/s12885-020-07683-7.

## Background

Advances in the detection and treatment of cancer have increased survival rates over the last decades. With improved survival rates, more patients will develop spinal metastases, which can lead to devastating consequences including progressive and unremitting pain, spinal instability and metastatic epidural spinal cord compression (MESCC) [1–3]. Spinal metastases may therefore significantly impair the patients’ health related quality of life (HRQOL). The treatment goal for patients with spinal metastases is to enhance HRQOL by suppressing symptoms for their remaining time. Radiotherapy has a central role in palliative treatment of uncomplicated painful spinal metastases, and aims to relieve pain and locally control the tumor. However, surgery may be required in case of mechanical pain, pathological fracture and/or neurological deficit caused by MESCC, with or without adjuvant radiotherapy [4].

A patient’s HRQOL is a subjective and multidimensional construct and hence influenced by satisfaction with current health status, usually encompassing physical, emotional, and social functioning [5]. Pre-treatment expectations have been shown to play an important role in post-treatment quality of life [6]. When pre-treatment expectations are met after treatment, patients are more likely to be satisfied and perceive their post-treatment health status as more favorable, resulting in a higher quality of life [7–9]. When expectations are not met after treatment, patients will be less satisfied and hence, quality of life will be lower [6, 9].

Little is known about expectations of patients with spinal metastases undergoing radiotherapy and/or surgery. Assuming that patients with spinal metastases share characteristics with patients who had spinal surgery for non-cancer related conditions and with advanced cancer patients, we performed a meta-aggregation to synthesize findings of published qualitative studies to explore patient expectations regarding treatment outcomes following spinal surgery and patient expectations regarding treatment outcomes in advanced cancer care. The objectives were to study patient expectations after treatment and to draw parallels with the metastatic spine population.

## Methods

We followed the Preferred Reporting Items for Systematic Reviews and Meta-Analyses (PRISMA) statement [10]. The protocol was prospectively registered in the PROSPERO database (CRD42020145151).

### Search strategy and selection criteria

Comprehensive searches were performed in the MEDLINE, EMBASE and PsycINFO databases. The search strategy was developed in close consultation with a university librarian. The search strategies can be found in Additional file 1. The search was restricted to articles in English or Dutch and published between 2000 and September 2019. Quantitative and qualitative studies that gave understanding of patients’ pre-treatment expectations regarding treatment outcomes were included. Studies were eligible when they included adult patients (> 18 years old) undergoing spinal surgery or with advanced cancer receiving palliative care. Two reviewers (AV and RG) independently screened titles and abstracts to identify articles requiring full-text review. The reference lists of included articles were searched for relevant articles. Next, full-text review was performed by two reviewers (DO and RG). If consensus was not reached, a third reviewer (AV) was consulted.

### Qualitative appraisal

Quality of the included studies was independently assessed by two reviewers (DO and RG). Qualitative studies were assessed using The Critical Appraisal Skills Programme (CASP) tool for qualitative studies and cohort studies to assess study aims, methods, design, recruitment, data collection and analysis, researcher-participants relationship, bias and confounding, ethics and reporting. The CASP tool for qualitative studies comprises 9 questions that are scored with ‘yes’, ‘no’ or ‘can’t tell’ to explore whether study results are valid and relevant [11]. Question 10 was adapted to assess the relevance of the study results for this review. The CASP tool for cohort studies was modified; question 3 was removed since there is no exposure that is relevant for this systematic review, and questions 5a and 5b were combined [12]. This resulted in 9 questions that are scored with ‘yes’, ‘no’ or ‘can’t tell’. Question 6 was only assessed when pre-treatment expectations and fulfillment of expectations were measured. Questions 7, 8 and 12 were used to assess the relevance of the study results for this review. Quality assessment was not used to exclude studies, but provided information about methodological rigor (i.e., appropriateness of the methods with regard to the study aims), credibility of findings (i.e., congruity between findings and supporting data), and robustness of included studies. This information was used as an indicator of the validity of the reported results and taken into account when interpreting the data.

### Data extraction

Data from the included studies were extracted by two authors (DO and RG) using a predefined data extraction form that included information on the study characteristics (study aim, study population, methodology, phenome of interest). Relevant results (i.e., findings) as well as accompanying illustrations (i.e., quotations, statements or other textual extractions) supporting the findings were extracted from the included studies. A level of credibility (unequivocal, credible or not supported) was assigned to each finding [13]. Discrepancies between reviewers were discussed with a third author (AV).

### Data analysis

Data synthesis of the extracted findings was done according to the meta-aggregation method developed by the Joanna Briggs Institute [14]. First, unequivocal and credible findings as extracted from the included studies were aggregated into categories based on similarity of outcomes. Next, these categories were combined based on similarity in concepts and outcomes, resulting in synthesized findings. Categories and synthesized findings were presented as statements, i.e., an overarching description that conveys the meaning of a set of categories or findings. Data synthesis was performed by one reviewer (RG). The data synthesis process was checked by, and discussed with a second reviewer (DO).

## Results

### Screening and search outcomes

**Fig. 1:**
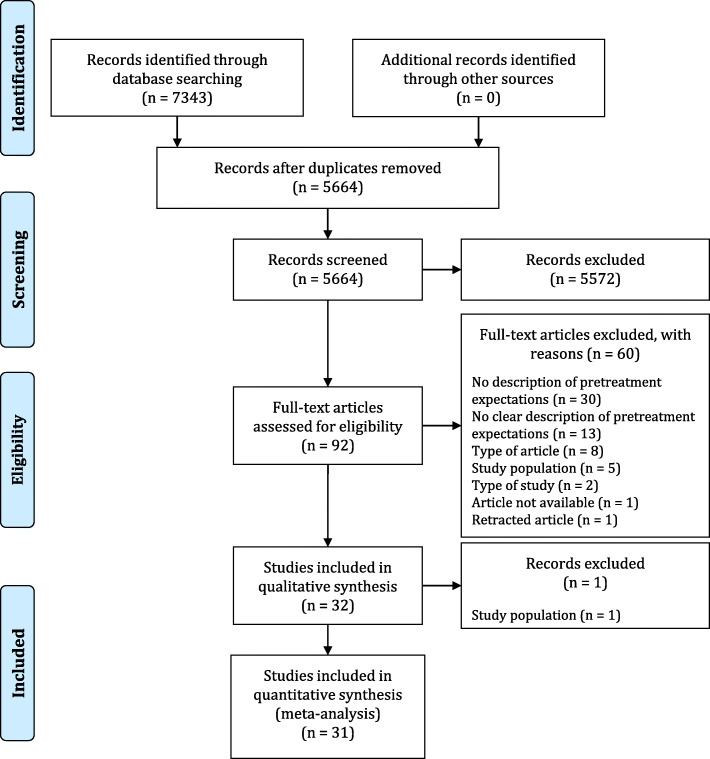
PRISMA flow diagram of the literature search and study selection

The search strategy yielded 7343 articles. After removing duplicates, 5664 titles and abstracts were screened, resulting in 92 articles for full-text review (Fig. 1). In total, 31 articles met the inclusion criteria.

### Included studies

The 31 studies were published between 2001 and 2019 (Table 1). Seventeen studies included patients undergoing spinal surgery and 14 studies included patients receiving advanced cancer care. In most studies, pretreatment expectations served as a determinant of a specific outcome such as post-operative pain, post-treatment fulfillment of expectations or satisfaction [6, 15, 17, 20–23, 25–27, 29, 30, 36–38, 41, 43]. In other studies, the main objective was to gain insight into expectations [16, 18, 19, 24, 28, 31–35, 39, 40, 42, 44]. Methods to measure expectations included (semi-structured) interviews, surveys and one study used audio-recordings of the consultation.

**Table 1:** Characteristics of the included studies

Author(s) and year of publication	Phenoma of interest	Study population	Mean age in years Sex, n (%) female	Method of data collection (expectations) Timing of measurement
**Patients undergoing spinal surgery**				
Accardi-Ravid et al. (2019) [15]	Preoperative and postoperative experiences of spine surgery including perioperative expectations, emotional experiences, long-term recovery, postoperative outcomes, interest in perioperative psychosocial interventions, and potential barriers and facilitators to participating in an intervention	14 patients who had spine surgery	57.3 (SD 15.7) 6 (42.9)	Semi-structured interviews 2–12 mo post-operatively
Lattig et al. (2013) [16]	Preoperative expectations of the short-term results in relation to pain, pain medication usage, sensory and motor function, and the ability to work, do household activities, and participate in sports Patient-surgeon discrepancies in expectations	241 patients (15-90y) undergoing spine surgery	62 (15) 133 (59)	Survey After preoperative consultation
Licina et al. (2012) [17]	Expectations of the surgical treatment (level of back and leg pain, and disability), and satisfaction with postoperative results	145 patients scheduled for primary, single-level surgery for degenerative lumbar spine conditions	54 (15) 54 (37)	Survey Preoperatively | 6 w/6 mo post-operatively
Mancuso et al. (2014) [18]	Long-term expectations of surgery as measured with the 20-item Hospital for Special Surgery, and associations with demographic, clinical, and psychological characteristics	150 patients (≥18y) scheduled for cervical spine surgery	55 (13) 59 (39)	Interview (about survey) Preoperatively
Mancuso et al. (2015) [19]	Associations between expectations as measured with the 20-item Hospital for Special Surgery, Lumbar/Cervical Spine Surgery Expectations Survey, and demographic, psychological, and clinical characteristics	420 patients (≥18y) scheduled for lumbar spine surgery	55 (15) 181 (43)	Interview (about survey) ±7 d post-operatively
Mancuso et al. (2016) [20]	Preoperatively stated expectations as measured with the 20-item Hospital for Special Surgery, Lumbar/Cervical Spine Surgery Expectations Survey and fulfillment of expectations post-operatively	Patients (≥18y) scheduled for lumbar (*n* = 366) or cervical (*n* = 133) spine surgery	Lumbar: 55 (SD 15) 157 (43) Cervical: 54 (SD 13) 51 (133)	Survey: ±7 d preoperatively Interview (telephone): 2 y post-operatively
Mancuso et al. (2017) [21]	Patient and clinical characteristics, including the 20-item Hospital for Special Surgery, Lumbar/Cervical Spine Surgery Expectations Survey, and pain improvement post-operatively	422 patients (≥18y) scheduled for lumbar spine surgery	56 (15) 190 (45)	Interview (structured): ±7 d preoperatively Interview (telephone): 2 y post- operatively
Mannion et al. (2009) [22]	Preoperative expectations as measured with a modified version of the “expectations scale” of the North American Spine Society (NASS) Lumbar Spine Questionnaire, changes in symptoms, and expectations being fulfilled	100 patients (>45y) with lumbar herniated disc or spinal stenosis, indication for decompression surgery without fusion	65 (SD 11) 33 (33)	Survey Preoperatively | 2 mo/12 mo post-operatively
McGregor et al. (2013) [23]	Preoperative expectations (e.g. state of health and their levels of back and leg pain) and importance of achieving this level of recovery, and satisfaction with the short and longer term outcome of surgery (in terms of pain and QoL)	316 patients scheduled for lumbar decompression or discectomy because of lateral nerve root compression or lumbar disc prolapse	range 53–55 170 (54)	Survey Preoperatively | 6 w/6 mo/12 mo post-operatively
Rehman et al. (2019) [24]	Preoperative expectations and the spine surgeon’s perspectives regarding treatment understanding, postoperative outcomes and information required for informed decision-making	12 patients (≥18y) with sciatica, scheduled for surgical decompression 6 surgeons	Patients: median 48 (range 24–74) 5 (43) Surgeons: median 50 (range 45–68)-	Semi-structured interviews 3–4 w after consultation, but preoperatively
Rönnberg et al. (2007) [25]	Relationships between baseline characteristics and expectations of surgical results (leg pain, back pain, sensibility, return to work), and satisfaction with provided care and given information	148 patients who had undergone surgery for a one-level disc herniation on the L4 –L5 or L5–S1 level	40 (range 18–66) 68 (46)	Survey Preoperatively | 2 y post-operatively
Saban and Penckofer (2007) [6]	Relationship between preoperative expectations of QoL, and postoperative perceived QOL and level of satisfaction and optimism	57 patients (≥18y) undergoing elective lumbar surgery for the first time for degenerative changes, herniated disks, or both	53.4 (SD 13.6) 30 (52.6)	Survey 2–14 d preoperatively | 3 m post-operatively
Soroceanu et al. (2012) [26]	Relationship between expectations as measured with the Musculoskeletal Outcomes Data Evaluation and Management System’s (MODEMS) expectations survey, and outcomes in the cervical versus the lumbar spine population	402 patients undergoing lumbar or cervical spine surgery	52.9 (15.2) 226 (56.3)	Survey Preoperatively | 6–12 w post-operatively
Toyone et al. (2005) [27]	Patient expectations of spine surgery including relief of leg pain, leg numbness and low back pain, and limitations in walking ability and activity of daily living, and the level of fulfillment of those expectations	Patients undergoing lumbar disc herniation (*n* = 49) or lumbar spinal stenosis (*n* = 49)	Disc herniation: 36 15 (31) Spinal stenosis: 67 22 (45)	Survey Preoperatively | 2 y post-operatively
van der Horst et al. (2019) [28]	Pre-operative expectations and perceptions, and post-operative experiences (e.g. limitations in daily functioning due to their back pain, other health complaints)	12 patients (≥18y) who had spinal fusion in last 6 mo	- 6 (50)	Survey 0–9 mo post-operatively
Yee et al. (2008) [29]	Expectations of surgery (regarding relief of back and leg pain, relief of numbness/weakness/instability, their ability to sleep, recreational and daily activities, and return to work), and its association with patient factors, baseline preoperative functional outcome scores and patient-reported improvements in functional outcome after surgery	143 patients undergoing decompression and/or spondylodesis (spinal fusion)	52 (range 18–84) 50%	Survey Preoperatively | 6 w/3 mo/6 mo/12 mo post-operatively
Yoo et al. (2019) [30]	Preoperative expectations and postoperative outcomes, and the effect on postoperative satisfaction	101 patients (>18y) undergoing 1- or 2-level minimally invasive spinal lumbar fusion surgery for degenerative pathology	57 43 (42.6)	Survey Preoperatively | 6 mo post-operatively
**Patients receiving advanced cancer care**				
Bergerot et al. (2019) [31]	Association between expectations of cure and QoL, anxiety and depression	60 patients with metastatic renal cell carcinoma, urothelial carcinoma or prostate cancer	65.1 (SD 13.1; range 31–91) 19 (31.7)	Survey Prior to immunotherapy and before counseling from their oncologist
Chen et al. (2013) [32]	Expectations about the goals of, and likelihood of cure from radiotherapy	384 patients with incurable lung cancer (stage IV or IIIB)	median: 63 154 (40)	Telephone survey 4–7 mo post-diagnosis
Chow et al. (2001) [33]	Illness understanding and expectations of palliative radiotherapy	60 patients with advanced cancer, referred for palliative radiotherapy	median 68 (range 46–90) 30 (50)	Survey Pre-consultation
Chow et al. (2007) [34]	Expected level of pain reduction, and influence of bone pain and having undergone the treatment on this expectation	217 patients (≥18y) with bone metastases, treated with palliative RT	median: 66 (range 28–88) 87 (40)	Interview Pre-radiation | 2 mo post-radiation
Craft et al. (2005) [35]	Understanding of the intent of their treatment (to monitor illness, improve QoL, control illness or cure illness) and that their illness was life-threatening, and sources of information	163 patients (>18y) with advanced cancer	- 89 (55)	Survey Week 1 and 12
Doyle et al. (2001) [36]	Patient expectations and perceptions of benefit	26 patients with recurrent or refractory advanced ovarian cancer, undergoing 2nd or 3nd line chemotherapy for	median: 55 26 (100)	Surgery Before chemotherapy
Friedlander et al. (2014) [37]	Symptom burden, and expected and perceived benefits of chemotherapy	126 patients with platinum resistant ovarian cancer and a life expectancy of > 3 mo, scheduled for chemotherapy	62 (range 30–89) 126 (100)	Survey < 2 w before chemotherapy | before each cycle | 4 w after 4th cycle
Gramling et al. (2016) [38]	Association between talking about expectations for length of life during inpatient palliative care consultations and rates of hospice enrollment	101 hospitalized patients (>21y) with metastatic cancer, referred for palliative care consultation	- 50 (43.5)	Audio-recording of the consultation
Mitera et al. (2012) [39]	Illness understanding and expectations of palliative radiotherapy	100 patients with advanced cancer, referred for a palliative radiotherapy consultation	66.2 (11.3) 44 (44)	Survey Pre-consultation / Post-consultation
Nowicki et al. (2015) [40]	Understanding and expectations of treatment, and socio-demographic factors	100 patients with lung cancer and a life expectancy of > 6 mo, undergoing palliative chemotherapy and an	63.1 (range 40–80) 34 (34)	Survey ?
Sjoquist et al. (2013) [41]	We explored associations among expected improvement, hope and indices of well-being, and perceived symptom benefits of chemotherapy	126 patients (≥18y) with recurrent and progressive ovarian cancer and a life expectancy of ≥3 mo	62.1 (9.8) 126 (100)	Survey Prior to chemotherapy | first four treatment cycles | 4 w post-treatment
Sze et al. (2006) [42]	Factors important in decision making for whole-brain radiation therapy for patients and caregivers	20 patients (or caregivers) with brain metastases within the past 2 mo, consideration of brain radiotherapy	median: 62 (range 50–72) 5 (25)	Open-ended, semistructured interviews Within 2 w after consultation
Visser et al. (2018) [43]	Satisfaction with therapy as measured with the Cancer Therapy Satisfaction Questionnaire (CTSQ), and patient- and treatment-related factors and patients’ feelings about adverse events	65 patients with locally advanced or metastatic stage IIIB/IV nonsquamous NSCLC, undergoing chemotherapy	62.1 (7.9) 50 (50)	Survey During 4th cycle of chemotherapy
Weeks et al. (2012) [44]	Expectation that chemotherapy might be curative and associated clinical, sociodemographic, and health-system factors, and physician communication	Patients with stage IV (i.e., metastatic) lung (*n* = 710) or colorectal cancer (*n* = 483), opted to receive chemotherapy	- Lung: 476 (67) Colorectal: 396 (82)	Structured interview 4–7 mo post-diagnosis

*d* days, *mo* months, *NSCLC* Non-small-cell lung carcinoma, *QoL* quality of life, *SD* standard deviation, *w* weeks, *y* years

The quality appraisal showed that the overall quality of the included studies was high (Table 2). However, most studies scored “no” on the item assessing the generalizability of the results because these studies enrolled patients from one (specialized) center, had a small sample size, or experienced selective drop-out [6, 18–21, 23, 26, 30–34, 36, 38, 43, 44].

**Table 2:** Quality appraisal of studies

**Qualitative studies**										
	**1. Clear statement of research aims?**	**2. Qualitative methodology appropriate?**	**3. Appropriate research design?**	**4. Appropriate recruitment strategy?**	**5. Data collection in a way that addressed research issue?**	**6. Relationship between researcher and participants adequately considered?**	**7. Ethical issues considered?**	**8. Sufficiently rigorous data analysis?**	**9. Clear statement of findings?**	**10. Results valuable for this review?**
Accardi-Ravid et al. (2019) [15]	Y	Y	Y	Y	Y	Y	N	Y	Y	Y
Rehman et al. (2019) [24]	Y	Y	Y	Y	Y	N	Y	Y	Y	Y
Sze et al. (2006) [42]	Y	Y	Y	Y	Y	?	Y	Y	Y	Y
van der Horst et al. (2019) [28]	Y	Y	Y	Y	Y	N	Y	Y	Y	Y
**Cohort studies**										
	**1. Clearly focused issue?**	**2. Acceptable recruitment?**	**3. Outcome accurately measured to minimise bias?**	**4. All confounding factors identified and taken into account?**	**5. Complete follow-up?**	**6. Follow-up long enough?**	**7. Do you believe the results?**	**8. Results generalizable?**	**9. Results fit with other available evidence?**	**10. Results valuable for this review?**
Bergerot et al. (2019) [31]	Y	Y	Y	Y	NA	NA	Y	N	Y	Y
Chen et al. (2013) [32]	Y	Y	Y	Y	N	NA	Y	N	Y	Y
Chow et al. (2001) [33]	Y	?	Y	Y	NA	NA	Y	N	Y	Y
Chow et al. (2001) [34]	Y	Y	Y	Y	N	N	Y	N	Y	Y
Craft et al. (2005) [35]	Y	Y	Y	Y	Y	Y	Y	Y	Y	Y
Doyle et al. (2001) [36]	Y	Y	Y	Y	Y	N	Y	N	Y	Y
Friedlander et al. (2014) [37]	Y	N	Y	Y	Y	Y	Y	Y	Y	Y
Gramling et al. (2016) [38]	Y	Y	N	N	Y	NA	Y	N	Y	Y
Lattig et al. (2013) [16]	Y	Y	Y	Y	Y	NA	Y	?	Y	Y
Licina et al. (2012) [17]	Y	Y	Y	Y	Y	Y	Y	Y	?	Y
Mancuso et al. (2014) [18]	Y	Y	Y	N	Y	NA	Y	N	Y	Y
Mancuso et al. (2015) [19]	Y	Y	Y	N	Y	Y	Y	N	Y	Y
Mancuso et al. (2016) [20]	Y	Y	Y	Y	Y	Y	Y	N	Y	Y
Mancuso et al. (2017) [21]	Y	Y	Y	N	Y	Y	Y	N	Y	Y
Mannion et al. (2009) [22]	Y	N	Y	N	Y	Y	Y	?	Y	Y
McGregor et al. (2013) [23]	Y	Y	Y	Y	Y	Y	Y	N	Y	Y
Mitera et al. (2012) [39]	Y	?	Y	Y	Y	Y	Y	Y	Y	Y
Nowicki et al. (2015) [40]	Y	Y	Y	Y	Y	NA	Y	Y	Y	Y
Rönnberg et al. (2007) [25]	Y	Y	Y	Y	Y	Y	Y	Y	Y	Y
Saban & Penckofer (2007) [6]	Y	Y	Y	Y	N	Y	Y	N	Y	Y
Sjoquist et al. (2013) [41]	Y	Y	Y	Y	Y	Y	Y	Y	Y	Y
Soroceanu et al. (2012) [26]	Y	Y	Y	Y	?	Y	Y	N	Y	Y
Toyone et al. (2005) [27]	Y	?	Y	Y	Y	Y	Y	Y	Y	Y
Visser et al. (2018) [43]	Y	Y	Y	Y	N	NA	Y	N	Y	Y
Weeks et al. (2012) [44]	Y	Y	Y	Y	NA	NA	Y	N	Y	Y
Yee et al. (2008) [29]	Y	Y	Y	Y	Y	Y	Y	Y	Y	Y
Yoo et al. (2019) [30]	Y	Y	Y	Y	Y	Y	Y	N	Y	Y

*N* no, *NA* not applicable, *Y* yes;? = can’t tell

### Synthesized findings

In total, 78 findings with 152 illustrations were extracted from the 31 included studies and assessed as credible or unequivocal. These were aggregated into 12 categories, which were combined to 3 synthesized findings.

#### Finding 1: the majority of patients expected improvement on several domains after treatment, but these expectations were often overly optimistic

Overall, patients scheduled for spinal surgery expected relief of their symptoms and improvement in physical functioning and undertaking daily activities after surgery (Table 3). However, many studies reported that patients had high and overly optimistic expectations of post-operative outcomes in terms of reduction in (back and leg) pain, symptom relief and better physical functioning [6, 16, 17, 22, 24, 25, 28, 31–33, 35, 36, 39, 40, 42–44]. For example, Mannion et al. concluded from their study on patients who had decompression spinal surgery that “*the expectations declared before surgery had been overly optimistic in approximately 30% to 40% patients for leg pain, back pain, walking capacity, independence, social function, and mental well-being, and in almost 50% patients for general physical capacity at home and work (general function) and sport.”* [22]

**Table 3:** Synthesized finding 1: The majority of patients expected improvement on several domains after treatment, but these expectations were often overly optimistic

Findings	Categories
**Patients undergoing spinal surgery**	**Patients had overly optimistic expectations about treatment outcomes.**
Patients planned for spinal surgery had much more optimistic expectations than their surgeons about their likely pain and activity level 3 months postoperative [16]. [U]	
Patients undergoing spinal surgery had high expectations of the treatment outcomes [17]. [C]	
Patients who had decompression surgery without fusion had overly optimistic expectations of the outcomes of surgery [22]. [U]	
Most patients scheduled for surgical decompression for sciatica expected complete recovery, including resolution of their back pain [24]. [U]	
Patients undergoing lumbar disc herniation surgery had high expectations [25]. [U]	
Patients who had elective lumbar surgery had fairly high expectations of their postoperative QOL [6]. [U]	
Patients who had undergone spinal fusion had overly optimistic expectations about recovery [28]. [U]	
**Patients receiving advanced cancer care**	
A substantial proportion of patients with metastatic cancer had the inaccurate expectation that cure after immunotherapy was likely [31]. [U]	
A large proportion of the patients with incurable lung cancer had inaccurate beliefs about the likelihood of cure from radiotherapy [32]. [U]	
About half of the patients with metastatic disease understood that their treatment was not curable, especially in patients without known brain metastases [33]. [U]	
Many patients with metastatic disease believed that radiation treatment could prolong their lives [33]. [U]	
Less than half of the patients with advanced cancer understood that their treatment was non-curative [35]. [U]	
Many patients with recurrent ovarian cancer thought that chemotherapy would have a moderate to high chance of curing their disease [36]. [U]	
After consultation with their radiation oncologist, about one-quarter of the patients undergoing palliative radiotherapy persist to believe their cancer is curable [39]. [U]	
After consultation with their radiation oncologist, about half of the patients undergoing palliative radiotherapy persist to believe that treatment will prolong their life [39]. [U]	
Almost half of the patients with lung cancer undergoing palliative chemotherapy were convinced that chemotherapy will cure them [40]. [U]	
Some patients receiving palliative radiotherapy still expect/hope that their tumor will go away [42]. [C]	
A substantial proportion of patients with advanced cancer had the inaccurate expectation that cure after chemotherapy was likely [43]. [U]	
Many patients with metastatic lung or colorectal cancer who had opted to receive chemotherapy had inaccurate expectations about the curative potential of chemotherapy [44]. [U]	
**Patients undergoing spinal surgery**	**Patients’ expectations exceeded the actual outcome.**
Expectations from patients who had undergone spinal surgery frequently exceeded the actual outcome [15]. [U]	
In more than half of the patients who had undergoing lumbar spinal surgery, expectations were not fulfilled [21]. [U]	
Patients who had undergone spinal surgery expected better outcomes than they achieved [23]. [U]	
Prepoperative expectations were higher than their fulfilled postoperative expectations in patients who had undergone lumbar surgery [6]. [U]	
Most expectations of patients who had undergone spinal fusion regarding the postoperative period were not fulfilled [28]. [U]	
In patients who had undergone spinal surgery, outcomes were not better than expected and even worse. However, some expectations were met [30]. [U]	
**Patients receiving advanced cancer care**	
Patients with advanced cancer expected that they would benefit more from chemotherapy than they actually did [37]. [U]	
Expected benefits from chemotherapy were higher than experienced benefits in patients who were treated with chemotherapy [41]. [U]	
**Patients undergoing spinal surgery**	**Patients expected improvement after treatment.**
Patients undergoing cervical spine surgery had diverse expectations that encompass improvement after surgery [18]. [U]	
Patients undergoing lumbar spine surgery expected that they would improve on many areas [19]. [U]	
Patients expected (much) improvement after decompression surgery [22]. [U]	
Patients planned for spinal surgery had optimistic expectations regarding post-treatment outcomes [26]. [U]	
Most patients undergoing spinal surgery expected that surgery will be successful and will relieve their symptoms [27]. [U]	
Patients undergoing spinal surgery had high expectations for relief of leg pain, improvement in sleep and return to household and recreational activities, and lower expectation for return to work-related activities [29]. [U]	
Preoperatively, patients undergoing spinal surgery expected significant improvements in postoperative outcomes [30].[U]	
**Patients receiving advanced cancer care**	
Patients with recurrent ovarian cancer have positive expectations of chemotherapy [36]. [C]	
Almost all patients with advanced cancer expected improvement from chemotherapy [41]. [U]	
**Patients undergoing spinal surgery**	**In the majority of the patients, some of the expectations were met.**
Expectations were reached in approximately half of the patients who had undergone spinal surgery [17]. [U]	
Almost all patients who had lumbar surgery had at least some of their expectations fulfilled [20]. [U]	
Almost all patients who had cervical surgery had at least some of their expectations fulfilled [20]. [U]	
Expectations were met in most of the patients who had undergone spinal surgery [27]. [U]	
Patient expectations regarding post-treatment outcomes for spinal surgery were met in the majority of patients [29]. [U]	
**Patients undergoing spinal surgery**	**Fulfillment of expectations differed between expectations.**
In patients who had lumbar surgery, the amount of improvement expected in pain was the expectation most often fulfilled [20]. [U]	
In patients who had lumbar surgery, return to work was the expectation least often fulfilled [20]. [U]	
In patients who had cervical surgery, the expected improvement in the ability to perform daily activities was the expectation most often fulfilled [20]. [U]	
In patients who had cervical surgery, return to work was the expectation least often fulfilled [20]. [U]	
Expectations regarding pain were most often fulfilled, while expectations regarding return to work and ability to work the least often fulfilled in patients who were planned for spinal surgery [26]. [U]	

*C* credible, *U* Unequivocal

Rehman et al. conducted interviews with surgeons and concluded the following: “*More often than not, surgeons reported that patients were overly optimistic about surgery, expecting complete recovery, including their back pain: “... often patients think their symptoms will go away 100%... so that’s the expectation I do try to dampen down, because it’s not realistic’”*“ [24]. This is line with the findings of Lattig et al. [16] They reported that patients consistently had higher expectations than their spine surgeon regarding back pain and functioning (i.e., activities at work, household activities, and sports).

From the studies that measured post-operative fulfillment of expectations, some studies reported (at least some) expectations regarding post-operative outcomes were met in the majority of the patients who had undergone spinal surgery [17, 20, 27, 29]. However, other studies reported that expectations were not fulfilled and exceeded actual outcomes [6, 15, 21, 23, 28, 30]. Van der Horst et al. reported that “*more than half of the participants (n = 7) had expected recovery to be easier or at least a more upwards trajectory instead of the struggle they experienced in reality.*” [28] Likewise, a patient in the study of Accardi-Ravid et al. said: “*The only thing that really surprised me—I was completely disabled as far as being mobile. And maybe they didn’t explain that to me, to my full benefit, that I would be completely disabled as far as walking and stuff like that. I thought I’d have more ambulatory ability... I had to really work on that as far as learning how to walk again and I had to use a walker and practice.*” [15] Mancuso et al. studied expectations of several domains in patients who had lumbar or cervical spine surgery, and concluded that expectations regarding pain were most often met, while return to work the least often [20].

Studies regarding expectations of patients with advanced cancer reported that patients expected improvement from chemotherapy (e.g., symptom relief and feeling better) [36, 41], but these expectations were overly optimistic [37, 41]. Most of these studies reported that patients with advanced cancer believe that their cancer is still curable and that the planned treatment (radiotherapy, chemotherapy or immunotherapy) is likely to cure their disease. For example, Sze et al. illustrated: “*Patients were able to acknowledge the terminal nature of their illness and still remain hopeful for a cure or remission. One lady said, “I’ll be around for another ten years. This brain tumor is going to be cured. It’s going to be shrunk to nothin”. However, when asked regarding the prognosis of her disease she said, “Well it’s not curable disease”. At times, there appeared to be a tension between a patient’s hope for cure and a suppressed realization of their true prognosis.*” [42]

#### Finding 2: patient counseling is important for patients’ understanding of disease and treatment

Information provided by spine surgeons was important for patients scheduled for spinal surgery (Table 4). Accardi-Ravid et al. reported that some patients who had undergone spinal surgery indicated that their surgeon managed their preoperative expectations by negotiating the treatment objectives and explaining the expected results to the patient [15]. Rehman et al. identified different methods for surgeons to improve patient understanding, e.g., stimulating further deliberation or calling the patient the night before the surgery [24]. However, other patients did not feel adequately prepared for surgery and “patients often mentioned vague, positive qualifiers in response to what they expected from surgery”, for example: “*To be honest, I thought I would go into—at least I felt like I was led to believe that—I would have the surgery, the pain would be relieved, and my neck would be stable and I could go on with my life.*” and “*I thought that I would just hang out in the hospital bed, take my drugs, be on my phone, and just watch TV.*” [15] Lattig et al. found patients and spine surgeons to have a different understanding of the terms associated with spinal disease, which may have resulted in overly optimistic patient expectations [16]. Rehman et al. concluded that providing an excessive amount of information was a barrier in disease and treatment understanding. Patients forgot information given by the spine surgeon or they were not able to process the information [24]. For example one spine surgeon expressed: “*In spite of explaining everything with the help of patient images and/or models, I am surprised how little they actually take home*.”

**Table 4:** Synthesized finding 2: Patient counseling is important for patients’ understanding of disease and treatment

Findings	Categories
**Patients undergoing spinal surgery**	**Pre-treatment consultation and patients’ understanding and expectations**
Some patients who had undergone spinal surgery did not feel adequately prepared for surgery [15]. [U]	
Patients who had undergone spinal surgery could often not describe preoperatively what they expect from the recovery process [15]. [C]	
The amount of information presented during the consultation with patients scheduled for surgical decompression for sciatica was excessive, and therefore, patients forget information that was given by the surgeon or don’t get the message [24]. [U]	
**Patients receiving advanced cancer care**	
Most patients referred for palliative radiotherapy reported that they were dissatisfied with the information from the referring physician [33]. [U]	
**Patients receiving advanced cancer care**	**Patients declared that they understood the treatment goal.**
About half of the patients with metastatic disease understood that palliative radiotherapy could relieve their symptoms [33]. [U]	
Advanced cancer patients with a low life expectancy had a clearer understanding of the treatment goal [35]. [U]	
After discussing with the radiation team, patients undergoing palliative radiotherapy have a better understanding of their cancer, intent of radiation treatment and are less worried about receiving treatment [39]. [U]	
The majority of patients with incurable lung cancer stated that chemotherapy aims to alleviate symptoms and improve quality of life [40]. [U]	
The majority of patients with lung cancer undergoing palliative chemotherapy stated that chemotherapy will prolong their life [40]. [U]	
The majority of patients with lung cancer declared that they are knowledgeable about palliative chemotherapy [40].[U]	
**Patients undergoing spinal surgery**	**Information needs of patients.**
Additional information was sought by patients who were planned for spinal surgery from external sources [24]. [U]	
**Patients receiving advanced cancer care**	
Patients receiving palliative radiotherapy (and their caregivers) have different information needs [42]. [U]	
**Patients undergoing spinal surgery**	**Patients’ disease and treatment understanding and expectations were dependent on their surgeon.**
Some patients who had undergone spinal surgery indicated that their surgeon prepared them preoperatively on what to expect [15]. [C]	
Patients and spine surgeons seem to have a different understanding of the terms associated with spinal diseases and hence different expectations, resulting in discrepancies between patient and surgeon expectations [16]. [C]	
When the spine surgeon is more experienced, the surgeon has lower expectations than their patient [16]. [C]	
There is variation in methods from spine surgeon to improve patients’ understanding [24]. [U]	
**Patients receiving advanced cancer care**	
Almost half of initial palliative care conversations with patients with metastatic cancer included at least one statement regarding expectations for the patient’s length of life [38]. [U]	

*C* credible, *U* Unequivocal

When asking patients with advanced cancer whether they understood the incurable nature of their disease, many studies reported that the majority of patients understood the treatment goals, i.e., palliation, symptom relief, and/or improvement of quality of life [33, 39, 40]. According to Sze et al., patients with advanced cancer had varying information needs for future decision making, e.g., about prognosis: “*… while some patients want more information, others have less need for knowledge to facilitate their decision making, preferring instead to defer to the knowledge of their doctors.*” [42]

#### Finding 3: patient expectations were influenced by various factors, including age, health condition and socioeconomic status

Multiple demographic, psychological and clinical characteristics were associated with patient expectations regarding treatment outcomes (Table 5). Younger patients undergoing spinal surgery who were physically and functionally more impaired had higher expectations (e.g., expected complete improvement) [18, 19]. One study found that better general health before surgery was associated with higher preoperative expectations [29], while another study found no association [27].

**Table 5:** Synthesized finding 3: Patient expectations were influenced by various factors, including age, health condition and socioeconomic status

Findings	Categories
**Patients undergoing spinal surgery**	**Characteristics that influenced expectations.**
Younger and more disabled patients who were scheduled for cervical spine surgery expected complete improvement in more areas [18]. [U]	
Multiple demographic, psychological, and clinical characteristics affect expectations of patients scheduled for lumbar spine surgery [19]. [U]	
Level of optimism and expectations were not correlated in patients undergoing elective lumbar surgery [6]. [U]	
Patients undergoing spinal surgery with better general health but poorer physical health reported higher expectations [29]. [U]	
**Patients receiving advanced cancer care**	
Patients with metastatic cancer with an older age, higher income, and lower rates of anxiety had more accurate expectations of cure from immunotherapy [31]. [U]	
Patients with incurable lung cancer who have inaccurate beliefs about radiotherapy also have inaccurate beliefs about chemotherapy [32]. [U]	
Especially patients with incurable lung cancer who were older, non-Caucasian, and who completed the survey by themselves (instead of by surrogates) were more likely to have inaccurate believes about radiotherapy [32]. [U]	
Patients with bone metastases with more pain expected a greater magnitude of pain reduction after palliative radiotherapy [34]. [U]	
Patients with lung cancer undergoing palliative chemotherapy and living in large towns and good economic status were more optimistic about the hope for cure, prolonging life and alleviation of symptoms [40]. [U]	
Hope and knowledge direct patient expectations of palliative radiotherapy [42]. [U]	
Especially non-Caucasian patients, patients with lung cancer, patients who received care outside an integrated health care network, and patients who reported higher scores for physician communication had more often inaccurate expectations [44]. [U]	
**Patients undergoing spinal surgery**	**Characteristics that were not related to expectations.**
Functional health status was not related to expectations of surgery in patients undergoing spinal surgery [27]. [U]	
**Patients receiving advanced cancer care**	
Disease characteristics and complaints had no impact on perceptions of cure in patients with metastatic disease [33]. [U]	
**Patients receiving advanced cancer care**	**Family and expectations.**
Caregivers wanted to maintain patients’ hope/expectations for cure after palliative radiotherapy [42]. [C]	

*C* credible, *U* Unequivocal

In patients with advanced cancer, one study found that older patients had more realistic expectations of cure [31]. In contrast, Chen et al. concluded that older patients were more likely to believe that they could be cured with palliative treatment [32]. A higher income was associated with more accurate and more optimistic expectations [31, 40]. Chow et al. reported that disease characteristics (e.g., Karnofsky performance status, site of primary cancer and metastases, and symptom distress) did not affect expectations of cure [33]. However, pain at diagnosis influenced expectations regarding post-treatment pain reduction [34].

## Discussion

This systematic review synthesized findings of expectations regarding treatment outcomes from patients undergoing spinal surgery for non-cancer related conditions and from patients with advanced cancer. Assuming that patients with spinal metastases share characteristics with these two populations, we studied patient expectations of these populations and draw parallels with the metastatic spine population. Based on these synthesized findings, we can conclude that patients who undergo spinal surgery and patients with advanced cancer tend to have overly optimistic expectations regarding treatment outcomes including pain and symptom relief, lower functional disability, (complete) recovery and prognosis. Discussing expected pain and symptom relief, recovery and prognosis before treatment may improve understanding of prognosis and promote and manage realistic expectations, which, in turn, may lead to better perceived outcomes and satisfaction.

Two previous systematic reviews concluded that higher preoperative expectations in patients undergoing spinal surgery predict higher post-operative satisfaction, improved functional outcomes and pain relief, but findings were not consistent [45–47]. In these reviews, no distinction was made between realistic and overly optimistic expectations. It may be that realistic positive expectations of post-operative improvement are associated with positive outcomes, but that overly optimistic expectations are associated with less favorable outcomes. Studies in patients undergoing other orthopedic surgical procedures (e.g., lower limb joint replacement) showed that not fulfilling pre-operative expectations was a strong predictor for dissatisfaction after surgery [48, 49]. Because patients with overly optimistic expectations are less satisfied with their post-operative health status, it is likely that they rate their quality of life lower. Saban et al. found that fulfillment of expectations was associated with higher quality of life [6].

Physicians have an important role in supporting patients to develop realistic expectations. Excessive amounts of information provided by physicians result in limited recall and diminished understanding of the disease and surgery [24]. In addition, discrepancies between patients and physicians in understanding of the medical terms associated with spinal disease might affect how patients appraise the information provided (e.g., what patients remember after consultation and which message they take home). This might result in overly optimistic expectations. Multiple studies in this review concluded that expectations of patients undergoing spinal surgery were too high and often not fulfilled after surgery. Given the impact of unfulfilled expectations on post-operative satisfaction and quality of life, it is important that physicians review patient expectations before surgery, and adjusts where needed.

Patients with advanced cancer often acknowledged that they understand the palliative treatment goal. Yet, most patients still expected that treatment will cure their disease. This contradiction raises the question as whether patients really understand the meaning and implication of palliation. Lay language and insuring that the patient understands the vocabulary used is critical. It is important that patients have realistic expectations of their prognosis because this will help them acknowledge their incurable disease status and engage in end-of-life planning discussions [50]. In addition, patients with unrealistic expectations of treatment outcomes may accept invasive and toxic treatments, which they would not have accepted when they had developed more realistic expectations. Discussing prognosis with the patient may help patients to develop a better understanding of the incurable nature of their disease [51].

Heterogeneity exists in characteristics influencing expectations. For example, physically more disabled patients were inclined to have more unrealistic expectations. Therefore, an individualized approach is essential in which the physician explores individual patient expectations and when unrealistic, subsequently tries to influence these expectations.

The incurable nature of metastatic spine disease may affect patient expectations after treatment which set them apart from the degenerative spine population. Although patients with metastatic spine disease often receive advanced cancer care, these patients may face unique challenges such as neurologic deficit and as such, their expectations may differ from those patients without spinal metastases. The treatment approach of patients with spinal metastases is often multidisciplinary (e.g., a medical or radiation oncologist, a spine surgeon, an oncology nurse), which may hamper consistent information disclosure. Therefore, more research is needed to gain insight into expectations of treatment outcomes in patients with spinal metastases and the best methods to instill appropriate or realistic expectations.

## Conclusions

Patients tend to have overly optimistic expectations regarding pain and symptom relief, recovery and prognosis. Pretreatment discussion about the expected pain and symptom relief, recovery and prognosis may improve understanding of prognosis, and promote and manage realistic expectations, which, in turn, may lead to higher satisfaction with the treatment outcome and hence a higher quality of life.

## Supplementary Information

**Additional file 1.** Search strategies.

## Data Availability

Data sharing is not applicable to this article as no datasets were generated or analysed during the current study.

## Abbreviations

CASP: Critical Appraisal Skills Programme
ENTREQ: Enhancing Transparency in Reporting the Synthesis of Qualitative Research
HRQOL: Health related quality of life
MESCC: Metastatic epidural spinal cord compression
