# Supplementary material for: Pre-treatment expectations of patients with spinal metastases: what do we know and what can we learn from other disciplines? A systematic review of qualitative studies
Source: BMC Cancer. 2020 Dec 9;20:1212. doi: 10.1186/s12885-020-07683-7 (PMC7724808; doi:10.1186/s12885-020-07683-7)
Supplement: Supplementary file 1 — Additional file 1. Search strategies. [file 12885_2020_7683_MOESM1_ESM.docx]

# Additional file 1 Search strategies

**PUBMED**

"Neoplasm Metastasis" [Mesh] OR neoplasm* [Title/Abstract] OR metastasis [Title/Abstract] OR metastases [Title/Abstract] OR metastatic [Title/Abstract] OR "incurable cancer*" [Title/Abstract] OR "advanced cancer*" [Title/Abstract] OR palliation [Title/Abstract] OR "palliative cancer" [Title/Abstract] OR "palliative radiotherapy" [Title/Abstract] OR "Spine" [Mesh] OR spine* [Title/Abstract] OR spinal [Title/Abstract] OR vertebra* [Title/Abstract] OR cervical [Title/Abstract] OR lumbar [Title/Abstract] OR thoracic [Title/Abstract] OR sacral [Title/Abstract] OR “back surgery” [Title/Abstract] OR decompressi* [Title/Abstract] AND (expectation* [Title/Abstract] OR "patient understanding" [Title/Abstract]) AND ("2000/01/01"[PDAT] : "2019/12/31"[PDAT])

**PsycINFO**

neoplasm*.mp,ab,ti. or (metastasis or metastases or metastatic or 'incurable cancer*' or 'advanced cancer*' or palliation or 'palliative cancer' or 'palliative radiotherapy').ab,ti. or spine.mp or (spine* or spinal or vertebra* or cervical or lumbar or thoracic or sacral or 'back surgery' or decompressi*).ab,ti. and (expectation or patient understanding).ab,ti.

limit [search] to yr="2000-Current"

**EMBASE**

‘Neoplasm’/exp OR (‘neoplasm*’ OR ‘metastasis’ OR ‘metastases’ OR ‘metastatic’ OR ‘incurable cancer*’ OR ‘advanced cancer*’ OR ‘palliation’ OR ‘palliative cancer’ OR ‘palliative radiotherapy’):ab,ti OR ‘Spine’/exp OR (‘spine*’ OR ‘spinal’ OR ‘vertebra*’ OR ‘cervical’ OR ‘lumbar’ OR ‘thoracic’ OR ‘sacral’ OR ‘back surgery’ OR ‘decompressi*’):ab,ti AND (‘expectation*’ OR ‘patient understanding’):ab,ti AND [2000-2019]/py AND [embase]/lim AND ('article'/it OR 'review'/it)
